# Self-Reported and FEMA Flood Exposure Assessment after Hurricane Sandy: Association with Mental Health Outcomes

**DOI:** 10.1371/journal.pone.0170965

**Published:** 2017-01-27

**Authors:** Wil Lieberman-Cribbin, Bian Liu, Samantha Schneider, Rebecca Schwartz, Emanuela Taioli

**Affiliations:** 1 Department of Population Health Science and Policy and Institute for Translational Epidemiology, Icahn School of Medicine at Mount Sinai, New York, New York, United States of America; 2 Department of Occupational Medicine, Epidemiology and Prevention, Hofstra Northwell School of Medicine, Manhasset, New York, United States of America; Columbia University, UNITED STATES

## Abstract

Hurricane Sandy caused extensive physical and economic damage; the long-term mental health consequences are unknown. Flooding is a central component of hurricane exposure, influencing mental health through multiple pathways that unfold over months after flooding recedes. Here we assess the concordance in self-reported and Federal Emergency Management (FEMA) flood exposure after Hurricane Sandy and determine the associations between flooding and anxiety, depression, and post-traumatic stress disorder (PTSD). Self-reported flood data and mental health symptoms were obtained through validated questionnaires from New York City and Long Island residents (N = 1231) following Sandy. Self-reported flood data was compared to FEMA data obtained from the FEMA Modeling Task Force Hurricane Sandy Impact Analysis. Multivariable logistic regressions were performed to determine the relationship between flooding exposure and mental health outcomes. There were significant discrepancies between self-reported and FEMA flood exposure data. Self-reported dichotomous flooding was positively associated with anxiety (OR_adj_: 1.5 [95% CI: 1.1–1.9]), depression (OR_adj_: 1.7 [1.3–2.2]), and PTSD (OR_adj_: 2.5 [1.8–3.4]), while self-reported continuous flooding was associated with depression (OR_adj_: 1.1 [1.01–1.12]) and PTSD (OR_adj_: 1.2 [1.1–1.2]). Models with FEMA dichotomous flooding (OR_adj_: 2.1 [1.5–2.8]) or FEMA continuous flooding (OR_adj_: 1.1 [1.1–1.2]) were only significantly associated with PTSD. Associations between mental health and flooding vary according to type of flood exposure measure utilized. Future hurricane preparedness and recovery efforts must integrate micro and macro-level flood exposures in order to accurately determine flood exposure risk during storms and realize the long-term importance of flooding on these three mental health symptoms.

## Introduction

Hurricane Sandy made landfall on the Northeastern United States on October 29^th^ 2012, resulting in 159 deaths and an estimated economic cost of $65 billion [1]. The unusual combination of the Hurricane, a nor’easter in early November, and coincidence with the full moon produced winds up to 90 mph and a storm-tide as high as 14 feet [1]. One of the main components of hurricane exposure is flooding. New York and New Jersey residents experienced extensive damage from inundation and high winds, as well as interruptions in basic water, electrical, and transportation services. Approximately 8.5 million customers lost power with outages lasting from weeks to months, and 50 deaths resulted from power outages in cold weather [1]. Over 650,000 houses and businesses were damaged or destroyed by Sandy, including 350,000 housing units in New Jersey and 300,000 homes destroyed in New York State, hundreds of buildings flooded in New York City (NYC), and 100,000 homes severely damaged or destroyed on Long Island (LI) [1].

Exposure to natural disasters and flooding causes physical and economic damage, but can also induce and exacerbate both immediate and long-term mental health concerns [2–7]. Generally, the severity of exposure and previous mental health problems are key predictors of distress following natural disasters [2, 8–10]. Direct consequences, such as experiencing personal injury, injury or death to a loved one, displacement from home, or loss of property, are risk factors for mental health problems [3, 10–12]. Natural disasters can also create stress from ongoing concerns such as lack of food and shelter, economic disruption, loss of employment, and interaction with insurance companies and social services [2, 6, 13–15]. These stressors result in higher rates of post-traumatic stress disorder (PTSD), depression, generalized anxiety, and perceived stress symptoms among victims following hurricanes compared to the general population [2, 7, 16–19].

The goals of the current study are to quantify the flood exposure experienced by NYC and LI residents during Hurricane Sandy and to compare self-reported flood exposure to macro-level FEMA reports of flood exposure in order to elucidate associations between type of flood exposure measure and mental health outcomes including anxiety, depression, and PTSD symptoms. This study is relevant to link the appropriate flood exposure measure to health effects and determine if any correlations exist between exposure measures, as well as delineate the impact of perception on mental health and understand which flood exposure measurements have the most public health relevance. We hypothesized a good concordance between the micro-level macro-level flood exposures and that flooding was positively associated with anxiety, depression, and PTSD symptoms, independently from the type of flood measure used.

## Materials and Methods

### Participants

The study population derives from two studies conducted in NYC/LI to assess the impact of Hurricane Sandy: the Leaders in Gathering Hope Together (LIGHT) Project (1/24/2013-2/25/2015) and Project Restoration (PR, 6/5/2014-1/9/2016). Approval for these studies was given by the internal review board of the Feinstein Institute for Medical Research at Northwell Health. In conjunction with community and governmental partners, the recruitment team traveled to libraries, community centers, senior centers, gyms and faith-based institutions across Queens, Staten Island, Nassau, and Suffolk in both heavily and less affected areas, and accepted all the volunteers who offered to participate in the study. Given that a convenience sampling strategy was used, the demographic characteristics of the participants were periodically compared to the census data for that county to assess the comparability of the convenience sample to the actual residents (S1 Table). Efforts were made to target recruitment to specific demographics in a particular region when a lack of comparability was noted. Differences can be attributed to oversampling in PR but generally, the study sample was congruous to the 2010 census data for the study’s target region.

While LIGHT was conducted in areas that were both heavily and mildly affected by Hurricane Sandy, recruitment for PR was specific to residents of the Rockaway Peninsula in Queens, which sustained heavy impact from Hurricane Sandy. Exclusion criteria were cognitive impairment that resulted in difficulty understanding consent or both non-English and non-Spanish speaking ability. At the time of this analysis, 1307 participants completed the survey, of which 670 and 637 were from LIGHT and PR, respectively. Of these, 11 reported a zip code outside the NYC / LI area, 30 did not provide an address, and 35 participants living in the NYC / LI area did not provide complete address information, leaving a sample of 1231 residents for the current study. Participants with missing data were not included in this analysis, although there was a very small amount of missing data due to staff direct oversight of participants while they were completing the survey. Any missing answers that were not caught during the initial completion were secondarily remedied by calling participants.

### Survey

Consented participants completed a 20-minute self-administered survey that queried participants’ demographics (e.g. age, sex, race), and medical and behavioral health status (e.g. smoking, current substance use, and current alcohol use). In addition, the survey utilized validated, psychometrically sound measures of mental health including anxiety, depression, and PTSD, which have been consistently documented to be associated with experiencing trauma [6, 20–25]. PTSD symptoms were assessed using the Post-Traumatic Stress Disorder Checklist-Specific (PCL-S), a 17-item standardized self-report measure reflecting DSM-IV PTSD symptoms that is tailored to be specific to a trauma, in this case Hurricane Sandy [20, 21, 26–28]. The Patient Health Questionnaire-4 (PHQ4) was used to assess symptoms of depression (first two items) and symptoms of generalized anxiety disorder (latter two items) [29]. The Cronbach’s alpha was 0.85 for anxiety and 0.84 for depression. Finally, participants completed questions regarding their mental health history before and after the hurricane.

### Main exposure variables

#### Self-reported flood data

As part of the survey, participants were asked to complete a checklist on Hurricane Sandy exposure items derived and adapted from previously used hurricane exposure tools [30, 31]. The presence and height of flooding were gathered from the overall hurricane exposure measure. Instances where participants recorded that there was no flooding in their home during Sandy (n = 63) were correspondingly assigned a flood height of 0 feet in order to be incorporated in a continuous measure of flood exposure. Instances where participants recorded flooding in their homes but did not record a numerical water height (n = 66) could not be included in analyses involving the continuous measure of flood exposure. A maximum water height of 15 feet reported on questionnaires was chosen as a cutoff to be included in this study to remove unreasonable flood heights. This choice excluded 4 participants.

#### Federal Emergency Management Agency (FEMA) flood data

Public macro-level flood data was obtained from the FEMA Modeling Task Force (MOTF) Hurricane Sandy Impact Analysis [32]. The approach utilized a combination of field teams and satellite imagery to provide a comprehensive assessment of Hurricane Sandy’s impact on New York, New Jersey, Connecticut, and Rhode Island. The New York State 3-meter spatial resolution storm surge product was downloaded and imported into our licensed copy of ArcGIS (version 10.3.1; ESRI, Redlands, CA) to provide water depth above ground in NYC and LI. Geocoded addresses were then overlaid on the FEMA flood layer to quantify the water height FEMA specified at every participant’s address.

### Geocoding

Street-level geocoding was performed in SAS (version 9.4; SAS Institute Inc., Cary, NC) using the proc-geocode procedure, relying on lookup reference datasets previously generated from U.S. Census Bureau TIGER/Line shapefiles [33, 34]. This process matches the street address, city, and zip code information in the survey dataset to the lookup dataset in order to produce a corresponding coordinate pair, as well as a numeric score indicating the quality of the match and what information (street address, city, zip code) was used to generate a match. On occasions where self-reported addresses were incomplete and could not be matched by SAS (n = 204), addresses were geocoded in small multiple batches over time in google maps together with non-participant addresses in order to protect the anonymity of participants.

### Outcome variables

Four mental health variables were considered in the current analysis based on the scores of mental health questionnaire. A cutoff score of 30 in the PCL-S response was used to indicate probable PTSD, and this measure was primarily used in the analyses. PTSD was subsequently assessed using the continuous form of the PCL-S and the DSM-IV algorithm [35] to discern if results changed under different criteria for determining PTSD. A mean score ≥2 on the first two items of the PHQ4 was calculated to establish a probable diagnosis of depression and a mean score ≥2 on the last two items of the PHQ4 was determined to establish a probable diagnosis of anxiety.

### Statistical analysis

Concordance between self-reported and FEMA flood exposures were assessed using a Wilcoxon rank sum test for flooding heights and the Kappa statistic for dichotomized (yes/no) flooding variables. Univariate comparisons in continuous and dichotomous flood exposures according to the dichotomized mental health variables were performed using Wilcoxon rank sum tests and Chi-square tests, respectively. Associations between flood exposure and mental health variables were investigated using logistic regression, adjusting for the following covariates: age (groups 18–25, 25–45, 45–65, and 65+ years), sex, race (white, non-white), education (≤ high school, > high school completed), existing mental health status (self-reported no mental health diagnoses, mental health diagnoses made prior to Hurricane Sandy, mental health diagnoses made after Hurricane Sandy, and mental health difficulties that existed both before and after Hurricane Sandy), time elapsed since Hurricane Sandy (11–18 months, 18–24 months, 24–30 months, and 30–39 months), and living in an apartment during Hurricane Sandy (yes/no). Existing mental health status was defined as reporting at least one of the following mental health difficulties: anxiety disorder, depression, PTSD, Schizophrenia, bipolar, substance/alcohol abuse, substance/prescription abuse, or other mental health problems. All significant findings are reported at the level of p < 0.05 (*) or p < 0.01 (**). Statistical analyses were performed using SAS (version 9.4; SAS Institute Inc., Cary, NC) and R (version 3.2.2; R Foundation for Statistical Computing, Vienna, Austria).

## Results

### Participants

The average age (±SD) of the participants was 45.5±19.8 years, and the median time elapsed since Hurricane Sandy was 20.16 months. Females represented 60.2% of the study population, the participants were equally distributed as white (53.1%) and nonwhite (46.9%), and 59.6% of participants had above a high school education (Table 1). Thirty-six percent of the responders reported having some degree of a history of mental health difficulties, with 13.9% reporting a history of mental health difficulties after, but not before, Hurricane Sandy. Based on the respective clinical cutoffs, 46% of participants indicated symptoms of probable anxiety, 37.5% indicated symptoms of probable depression, and 32.0% indicated symptoms of probable PTSD.

**Table 1:** Description of the study population.

VARIABLES (N/missing)		Frequency N (%)	SR Flooding YesN (%)	FEMA Flooding Yes N (%)	SR flood height (ft) (mean±SD)	FEMA flood height (ft) (mean±SD)
**Sex (1225/6)**	**Male**	476 (38.9)	144 (36.5)	188 (38.0)	1.21±2.42	1.28±1.94
	**Female**	749 (61.1)	250 (63.5)	307 (62.0)	1.27±2.51	1.43±2.02
**Race (1209/22)**	**White**	643 (53.2)	197 (50.1)	234 (47.8) *	1.38±2.73	1.22±1.94 *
	**Non White**	566 (46.8)	196 (49.9)	256 (52.2)	1.12±2.18	1.54±2.04
**Education (1183/48)**	**≤ High School**	469 (39.6)	159 (41.6)	239 (49.9) **	1.28±2.51	1.74±2.08 **
	**> High School**	714 (60.4)	223 (58.4)	240 (50.1)	1.24±2.47	1.13±1.9
**Age (year)s (1220/11)**	**18–25**	229 (18.8)	45 (11.5) **	52 (10.5) **	0.56±1.59 **	0.71±1.53 **
	**25–45**	368 (30.2)	127 (32.3)	156 (31.6)	1.28±2.42	1.43±2.00
	**45–65**	398 (32.6)	163 (41.5)	209 (42.4)	1.76±2.91	1.89±2.21
	**65+**	225 (18.4)	58 (14.7)	76 (15.5)	0.97±2.27	1.02±1.71
**Existing mental health status (1216/15)**	**No**	782 (64.3)	226 (57.5) **	287 (58.3) **	1.11±2.32 **	1.26±1.95 **
	**Prior to Sandy**	189 (15.5)	48 (12.2)	64 (13.0)	0.88±2.14	1.01±1.76
	**After Sandy**	161 (13.2)	84 (21.4)	93 (18.9)	2.02±2.83	1.99±2.11
	**Prior and After**	84 (6.9)	35 (8.9)	48 (9.8)	1.94±3.45	2.02±2.27
**Elapsed time (months) since Sandy (1231/0)**	**11–18**	488 (39.6)	149 (37.8)	167 (33.6) **	1.27±2.55	1.18±1.90
	**18–24**	372 (30.2)	116 (29.4)	155 (31.2)	1.17±2.40	1.36±1.96
	**24–30**	153 (12.4)	51 (12.9)	56 (11.3)	1.32±2.31	1.31±2.15
	**30–39**	218 (17.7)	79 (20.0)	119 (23.9)	1.26±2.57	1.87±2.07
**Anxiety score (1227/4)**	**<2**	667 (54.4)	183 (46.3) **	264 (53.2)	1.08±2.26 **	1.35±1.96
	**≥2**	560 (45.6)	212 (53.7)	232 (46.8)	1.46±2.71	1.40±2.03
**Depression score (1227/4)**	**<2**	776 (63.2)	213 (53.9) **	292 (58.9) *	1.08±2.33 **	1.29±1.94 *
	**≥2**	451 (36.8)	182 (46.1)	204 (41.1)	1.55±2.71	1.50±2.08
**PTSD score (1221/10)**	**<30**	835 (68.4)	208 (52.7) **	268 (54.1) **	0.93±2.20 **	1.10±1.85 **
	**≥30**	386 (31.6)	187 (47.3)	227 (45.9)	1.97±2.91	1.96±2.16

* p <0.05,

** p < 0.01. Chi-square tests were used on dichotomous flood exposures and variables, Wilcoxon rank sum tests were used on continuous flood exposures and variables. SR = self-reported.

### Comparison in assessment of flood exposure

**Fig 1:**
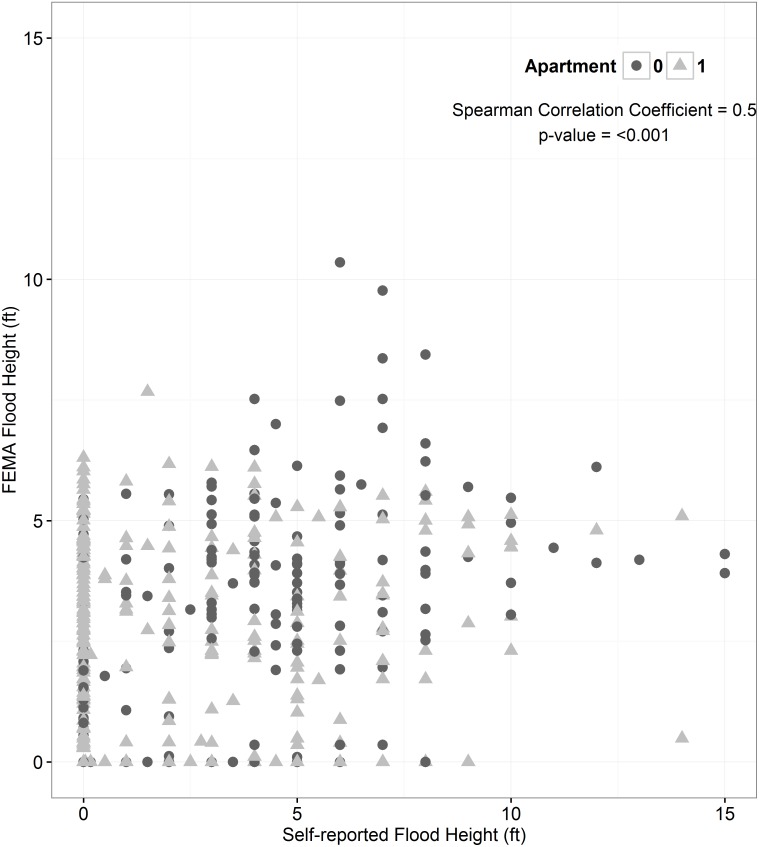
Correlation between FEMA (y) and self-reported (x) flood heights according to living in an apartment (triangles) or not (circles).

There was a moderate agreement between self-reported and FEMA flooding (Kappa statistic 0.46) and continuous (Spearman’s correlation coefficient 0.50; Fig 1) measures of flood exposure. A statistically significant difference (p < 0.001) was found between self-reported continuous flood heights (mean±SD: 1.24±2.48 feet, range: 0.0–15.0 feet) and FEMA-reported continuous flood heights (mean±SD: 1.37±1.99 feet, range: 0.0–10.4 feet). Flooding was self-reported and recorded by FEMA in 23.6% of cases, while agreement between the two measures on no flooding was 51.1%. Flooding was self-reported but not recorded by FEMA in 8.5% of cases, while flooding was not self-reported but indicated by FEMA in 16.8% of cases. In this last instance, 84% of people (173/207; 83.6%) resided in an apartment (no flooding reported) while the mean flood height reported by FEMA was 3.2 feet.

**Fig 2:**
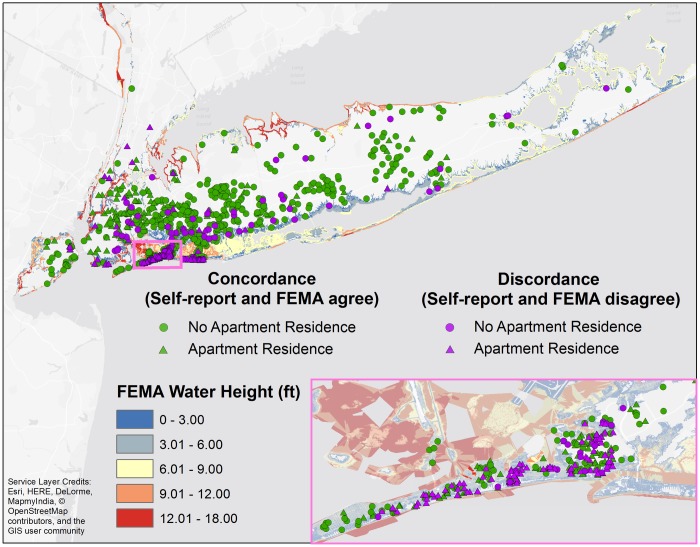
Geographic distribution of concordance (green) and discordance (purple) in flooding (yes/no) between self-reported and FEMA flooding measures in NYC and LI. Data were classified by living in apartments (triangles) and not (circles) and were overlaid on the FEMA MOTF flood height layer.

Instances of concordance and discordance between self-reported flooding and FEMA-reported flooding were overlaid on the FEMA MOTF water depth above ground layer (Fig 2). The most concordance (green) resided in the interior of NYC / LI, while the greatest areas of discordance (purple) were concentrated in the Rockaway Peninsula and Long Beach, and were especially present among those living in apartments (triangles).

### Univariate analyses of flood exposure and mental health

**Fig 3:**
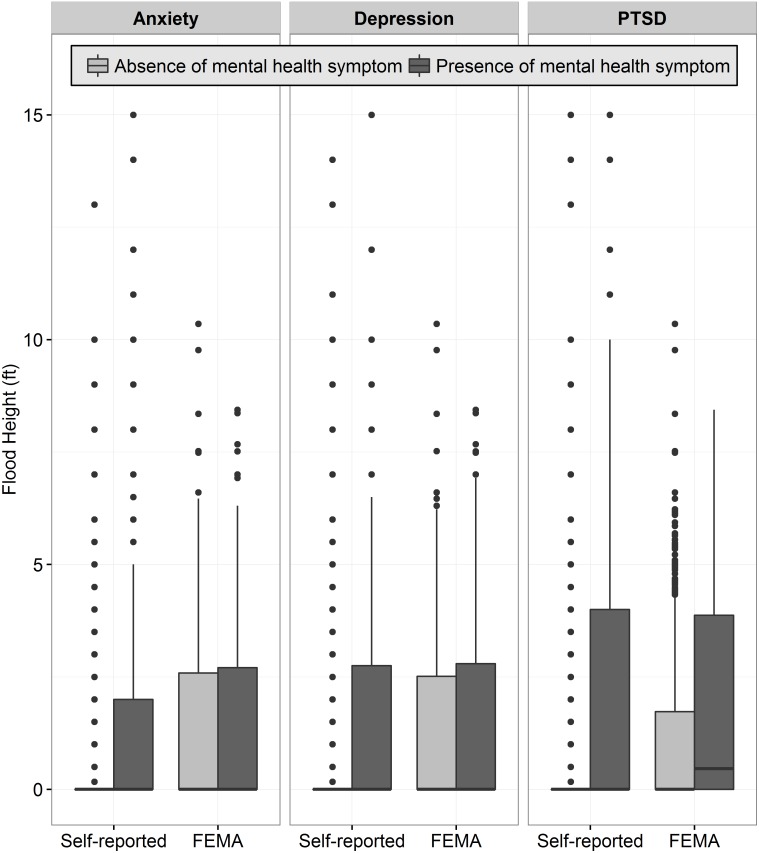
Self-reported and FEMA-reported flood heights and presence of mental health symptoms. * p < 0.05, ** p < 0.01. Wilcoxon rank sum tests were used to compare flood heights and the absence/presence of mental health symptoms within self-reported and FEMA-reported flood exposures.

Significant differences in flood heights were observed between self-reported flood exposure and the presence of anxiety, depression, and PTSD, as well as between FEMA flood heights and PTSD symptoms (Table 1, Fig 3). Utilization of the dichotomous self-report flooding measure, as compared to the dichotomous FEMA flooding indicator, resulted in a stronger association between flooding and anxiety, depression, and PTSD symptoms (Table 1).

### Multivariable analyses of flood exposure and mental health

#### Anxiety

**Fig 4:**
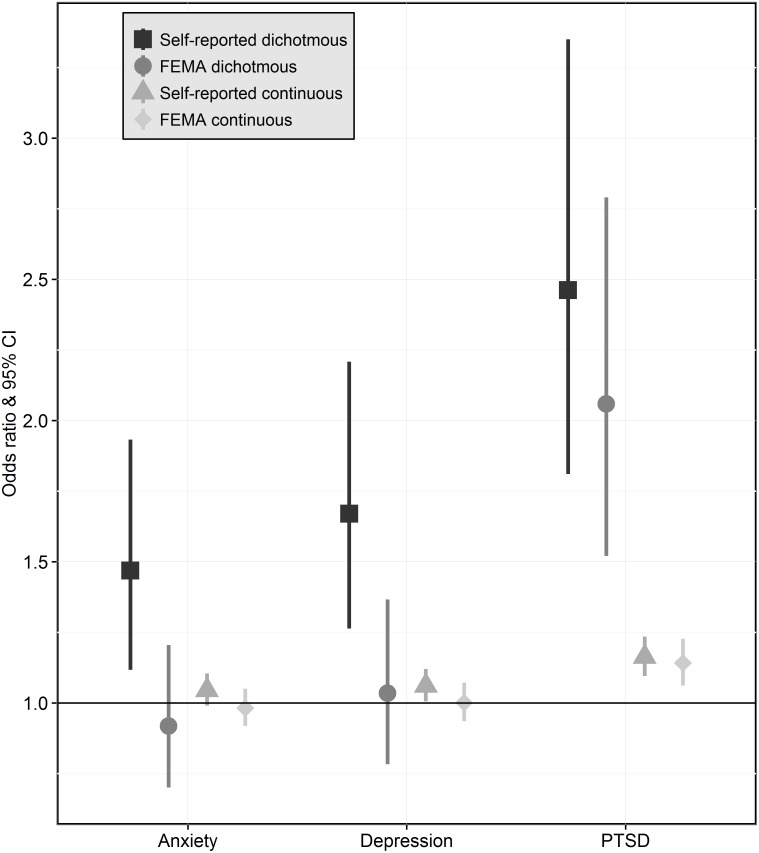
Associations between flood exposure measurements [self-reported / FEMA-reported, dichotomous (flooding yes/no) / continuous (feet)] and mental health (anxiety, depression, PTSD). The model was adjusted for age, gender, race, education, existing mental health status, elapsed time since Hurricane Sandy, and living in an apartment.

Anxiety was statistically significantly associated with dichotomous (OR_adj_: 1.5, 95%CI [1.1–1.9]) and continuous (OR_adj_: 1.0 [1.0–1.1]) self-reported flood exposures but not with either FEMA flood exposure (Fig 4). When dichotomous self-reported flood exposure was used in the model, female gender (OR_adj_: 1.4 [1.1–1.9]), history of mental health difficulties prior to Sandy (OR_adj_: 3.2 [2.2–4.5]), history of mental health difficulties after Sandy (OR_adj_: 4.8 [3.2–7.1]), and a history of mental health difficulties both prior and after Sandy ([OR_adj_: 6.9 [3.9–12.1]) were positively associated with anxiety; increasing age (45–65 years: OR_adj_: 0.7 [0.5–0.9]; 65+ years: OR_adj_: 0.5 [0.4–0.8]) and white race (OR_adj_: 0.7 [0.5–0.9]) were inversely associated with anxiety. The models with continuous self-reported, dichotomous FEMA, and continuous FEMA flood exposures yielded similar associations. These associations persisted when the PHQ-4 continuous anxiety score was used as the outcome (data not shown).

#### Depression

Depression was statistically significantly associated with dichotomous (OR_adj_: 1.7 [1.3–2.2]) but not with continuous self-reported flood exposure; no association was found with either FEMA flood exposure measure (Fig 4). When dichotomous self-reported flood exposure was used in the model, a history of mental health difficulties prior to Sandy (OR_adj_: 2.9 [2.0–4.1]), after Sandy (OR_adj_: 4.6 [3.1–6.7]), and both prior and after Sandy (OR_adj_: 4.4 [2.7–7.3]) were positively associated with depression symptoms. Similar results were obtained when continuous self-reported, dichotomous FEMA, and continuous FEMA flood exposures were used in the model. These associations persisted when the PHQ-4 continuous depression score was used as the outcome (data not shown).

#### PTSD

PTSD symptoms were statistically significantly associated with dichotomous (OR_adj_: 2.5 [1.8–3.4]) and continuous self-reported flood exposures (OR_adj_: 1.2 [1.1–1.2]), as well as dichotomous (OR_adj_: 2.1 [1.5–2.8]) and continuous (OR_adj_: 1.1 [1.1–1.2] FEMA flood exposures (Fig 4). When dichotomous self-reported flood exposure was used in the model, middle age (45–65 years: OR_adj_: 1.9 [1.3–2.7]), history of mental health difficulties prior to Sandy (OR_adj_: 2.6 [1.7–3.8]), history of mental health difficulties after Sandy (OR_adj_: 9.6 [6.2–14.8]), history of mental health difficulties both prior and after Sandy (OR_adj_: 6.5 [3.8–11.0]), shorter time since Hurricane Sandy (OR_adj_: 1.7 [1.2–2.5]), and living in an apartment (OR_adj_: 1.7 [1.2–2.4]) were positively associated with PTSD symptoms; education (OR_adj_: 0.6 [0.4–0.8]) was inversely associated with PTSD symptoms. The strength and direction of the covariates were similar under continuous self-reported, dichotomous FEMA, and continuous FEMA flood exposures. These associations persisted when the DSM-IV criteria were used to assess PTSD (dichotomous self-reported OR_adj_: 3.87 [2.56–5.85], continuous self-reported OR_adj_: 1.16 [1.08–1.24], dichotomous FEMA OR_adj_: 2.24 [1.48–3.81], continuous FEMA OR_adj_: 1.15 [1.05–1.27]). When the continuous PTSD score from the PCL-S was used as the outcome, PTSD was significantly associated with continuous self-reported (β: 0.9571, p < 0.0001) and continuous FEMA (β: 0.7639, p < 0.0001) flood exposures but not with dichotomous self-reported (β: 6.82, p 0.7358) or dichotomous FEMA (β: 3.94, p 0.7417) flood exposures.

## Discussion

This study is the first of its kind to compare macro and micro Hurricane Sandy flood exposure assessments and to examine the associations between flooding and mental health outcomes (anxiety, depression, PTSD) according to the type of flood exposure measurement used. Results indicated that there are significant discrepancies between self-reported and FEMA flood exposure data and that the associations between flooding and mental health outcomes vary according to the type of flood exposure measure utilized in the model, with self-perception of exposure being a stronger predictor of mental health outcomes than FEMA objective measurements. This finding has important public health implications as it underlines the importance of perception of danger, rather than objective measures, in determining mental health symptoms.

Flood exposure is an important component of hurricane exposure, but no study to date has compared the concordance of micro and macro-level flood exposures. Several studies analyzing macro-level exposure after Sandy have examined the accuracy of FEMA’s flood inundation products relative to previous FEMA predictive flood-risk maps [36, 37], with variability in the accuracy of predictions due to both the rarity of a storm like Sandy, as well as the geographic variability of newer maps and technology to predict the hurricane (43). Our comparisons of macro and micro-level flooding showed that 312 participants (25% of the sample) disagree with FEMA’s estimates. Discordance can be partially explained by observing that 84% of participants who recorded no flooding, but were classified as having flooding from FEMA’s estimate, live in apartments. Participants on other than the ground floor could conceivably have no flooding in their apartment, but have flooding in their building. In this case, FEMA’s reports of flooding in the building may not coincide with the participant’s perception, as their individual apartment unit was not flooded. Discordance can also partially be explained by the inherent limitations of SAS geocoding. The SAS geocode procedure is limited to street level only, in that latitude and longitude coordinates produced for an address are located in the street, as opposed to inside a building, thus not accounting for differences in water height between the two locations.

Previous research has partially integrated flooding into a mental health assessment by sampling NYC residents after Hurricane Sandy according to amount of area flooded under FEMA estimates; however hurricane exposure was qualitatively assessed and lacked a quantitative measure of individual flood exposure [24]. Other quantitative assessments of self-reported flood exposure during Sandy have not examined the specific role of flooding on anxiety, depression, and PTSD [38], nor compared individual exposure to macro-level exposures [39].

The present analysis highlights that associations between flood exposure and mental health outcomes differ according to the type of exposure measurement used, with stronger associations in micro-level (i.e. self-reported) than macro (i.e. FEMA) exposure measures. It is plausible that participants over-reported flooding compared to a macro-level estimate, but the veracity of individual estimates are arguably less important than individual’s perception of flood height, as perceptions may influence mental health status [6, 12, 40, 41]. The subjective perception of danger may also play a role in the continued amplification of the perceived threat, as previously discussed in research following the 2004 Indian Ocean Tsunami (45, 46). The importance of self-perception of flooding underscores the public health importance of accurate exposure assessments in order to determine individual mental health risk in populations exposed to natural disasters. In addition, these results shed light on the complex relationship between exposure, personal socio-economic factors, and system-level organizational elements that can operate together as negative amplifiers of the individual perception of exposure [2, 6, 8, 25, 42, 43]. The study also suggests that a simple self-measure of exposure such as flooding may be perceived differently according to other unmeasured confounding factors that act at a very personal level such as fear of job loss, property damage, or family displacement [13–15, 44, 45]. Thus, flooding acts on multiple pathways among mental health determinants that unfold over months to years after flood waters recede, highlighting the need for longitudinal studies capturing the long-term mental health effects of flooding [5, 8, 25, 43, 46]. This research is especially imperative given the increasing prevalence of higher intensity hurricanes due to climate warming [47, 48].

This study has some limitations. Convenience sampling was utilized in Project LIGHT and Project Restoration, allowing for potential sampling bias and skewed estimates of mental health conditions. It is possible that people who had more exposure during Sandy or were looking for help to recover from Sandy could be more likely to participate, potentially increasing the prevalence of mental health symptoms in the cohort. Financial incentives for completing the questionnaire could have also influence the participation of residents from low socioeconomic communities. To limit the bias associated with convenience sampling, recruitment efforts were targeted to ensure the study sample reflected the demographic distribution of the recruitment area and provided variability in geographic hurricane exposure. As in previous studies, mental health difficulties were evaluated using brief, self-report measures that are not diagnostic in nature, but result in assessments of symptomatology. These measures however have repeatedly demonstrated validity and reliability and are consistently correlated with more comprehensive diagnostic tools assessing their corresponding mental health disorder [26, 27, 29, 49, 50].

## Conclusion

This study emphasizes the strong effect of the type of exposure measure used in identifying the associations between flood exposure and mental health outcomes. The importance of perception was highlighted by the fact that self-reported dichotomous flooding resulted in significant associations with all mental health outcomes, while dichotomous FEMA flooding was only associated with PTSD symptoms. While macro-level FEMA flood data is a relatively less expensive and faster way to provide exposure estimates spanning larger geographic areas affected by Hurricane Sandy than micro-level estimates from cohort studies, macro-level exposure estimates may underestimate the full mental health impact of the hurricane. Future efforts to assess mental health needs in affected populations must therefore incorporate both micro and macro-level flood exposures and integrate them in order to produce the most accurate evaluations. Flooding is a unique aspect of natural disasters since it can be prepared for through flood-prevention measures, and thus hurricane preparedness and recovery efforts must accurately determine flood exposure risk during storms, as well as realize the long-term importance of flooding on mental health concerns.

## Supporting Information

S1 Table Study Sample Demographics compared to the US Census. (DOCX)
