# Supplementary material for: Self-Reported and FEMA Flood Exposure Assessment after Hurricane Sandy: Association with Mental Health Outcomes
Source: PLoS One. 2017 Jan 27;12(1):e0170965. doi: 10.1371/journal.pone.0170965 (PMC5271356; doi:10.1371/journal.pone.0170965)
Supplement: S1 Table — (DOCX) [file pone.0170965.s001.docx]

| **Demographics** | **Long Island, Queens & Staten Island (2010 Census)** | **Study Sample** |
| --- | --- | --- |
| **Population** | N=5,532,334 | N=1231 |
| **Population >=18** | % | % |
| **Population over Age 65** | 17.5 | 18.4 |
| **Females** | 51.4 | 61.1 |
| **Black or African American alone** | 13.3 | 38.5 |
| **American Indian and Alaska Native alone** | 0.5 | 0.5 |
| **Asian alone** | 12.6 | 1.7 |
| **Native Hawaiian and Other Pacific Islander alone** | 0.2 | 0.3 |
| **Two or more Races** | 3.3 | 6.8 |
| **Hispanic or Latino** | 20.5 | 18.8 |
| **White alone, not Hispanic or Latino** | 51.7 | 36.8 |
| **Average persons per household (2009-2013)** | 2.91 | 3.1 |
| **> High school graduate, over Age 25 (2009-2013)** | 85.9 | 83.4 |
| **> Bachelor's degree, over Age 25 (2009-2013)** | 33.5 | 32.7 |
| **Persons without health insurance, under Age 65** | 14.1 | 11.8 |
